# Patient partner perspectives on compensation: Insights from the Canadian Patient Partner Survey

**DOI:** 10.1111/hex.13971

**Published:** 2024-01-15

**Authors:** Roma Dhamanaskar, Laura Tripp, Meredith Vanstone, Carolyn Canfield, Mary Anne Levasseur, Julia Abelson

**Affiliations:** ^1^ Health Policy PhD Program, Faculty of Health Sciences McMaster University Hamilton Ontario Canada; ^2^ Public and Patient Engagement Collaborative McMaster University Hamilton Ontario Canada; ^3^ Department of Health Research Methods, Evidence, and Impact McMaster University Hamilton Ontario Canada; ^4^ Department of Family Medicine McMaster University Hamilton Ontario Canada; ^5^ Centre for Health Economics and Policy Analysis McMaster University Hamilton Ontario Canada; ^6^ Patient Advisors Network Canada; ^7^ Department of Family Practice University of British Columbia Vancouver British Columbia Canada; ^8^ Interdisciplinary Chair in Health and Social Services for Rural Populations Université du Québec à Rimouski Rimouski Quebec Canada; ^9^ Canada Research Chair in Partnership with Patients and Communities Montreal Quebec Canada

**Keywords:** compensation, health policy, healthcare, patient engagement, patient involvement, patient partnership

## Abstract

**Introduction:**

There is a growing role for patients, family members and caregivers as consultants, collaborators and partners in health system settings in Canada. However, compensation for this role is not systematized. When offered, it varies in both type (e.g., one‐time honorarium, salary) and amount. Further, broad‐based views of patient partners on compensation are still unknown. We aimed to describe the types and frequency of compensation patient partners have been offered and their attitudes towards compensation.

**Methods:**

This study uses data from the Canadian Patient Partner Study (CPPS) survey. The survey gathered the experiences and perspectives of those who self‐identified as patient partners working across the Canadian health system. Three questions were about compensation, asking what types of compensation participants had been offered, if they had ever refused compensation, and whether they felt adequately compensated. The latter two questions included open‐text comments in addition to menu‐based and scaled response options. Basic frequencies were performed for all questions and open‐text comments were analyzed through inductive qualitative content analysis.

**Results:**

A total of 603 individuals participated in the CPPS survey. Most respondents were never or rarely offered salary (81%), honorarium (64%), gift cards (80%) or material gifts (93%) while half were offered conference registration and expenses at least sometimes. A total of 129 (26%) of 499 respondents reported refusing compensation. Of 511 respondents, half felt adequately compensated always or often, and half only sometimes, rarely or never. Open‐text comments revealed positive, ambivalent and negative attitudes towards compensation. Attitudes were framed by perceptions about their role, sentiments of giving back to the health system, feelings of acknowledgement, practical considerations, values of fairness and equity and accountability relationships.

**Conclusions:**

Our findings confirm that compensation is not standardized in Canada. Half of survey respondents routinely feel inadequately compensated. Patient partners have diverse views of what constitutes adequate compensation inclusive of personal considerations such as a preference for volunteering, and broader concerns such as promoting equity in patient partnership. Organizations should attempt to ensure that compensation practices are clear, transparent and attentive to patient partners' unique contexts.

**Patient Contribution:**

Two patient partners are members of the CPPS research team and have been fully engaged in all study phases from project conception to knowledge translation. They are co‐authors of this manuscript. The survey was co‐designed and pilot tested with patient partners and survey participants were patient partners.

## 1 INTRODUCTION

Patients, family members and caregivers are increasingly sought as consultants, collaborators and partners in health system settings in Canada.^1^, ^2^, ^3^, ^4^, ^5^ We now see these individuals (herein referred to as ‘patient partners’) being directly embedded within health care organizations, government bodies or research groups to provide sustained input on their objectives and operations.^6^, ^7^ Historically, patient partners have been volunteers in these roles, reflecting a long tradition of volunteerism in the patient engagement movement and parallel movements in hospitals such as hospital volunteer associations.^8^, ^9^ However, as the patient partner role continues to expand and intensify, calls for adequate compensation of patient partners to recognize their time and contributions have followed.^10^ These calls include advocacy from patient partners themselves and statements from organizations and funding bodies seeking to standardize compensation.^10^, ^11^, ^12^, ^13^, ^14^

Some Canadian organizations have responded to these calls by creating compensation guides to inform practice.^15^, ^16^, ^17^, ^18^ While all guides suggest that compensation is important to acknowledge the time, skill and expertise of patient partners, most do not give standardized recommendations about procedures and rates of compensation. Indeed, many Canadian organizational compensation guides suggest that compensation should depend on the level of commitment, complexity of work and degree of participation that is required for the project or offered by the patient partner.^15^, ^16^, ^17^, ^18^ Salary, one‐time payments, gift cards and honoraria have all been suggested as forms of compensation for Canadian patient partners. Importantly, reimbursement of expenses (e.g., for travel) is not, by definition, compensation but is a common practice to ensure that patient partners do not incur any costs for participation.^10^ Compensation goes above and beyond reimbursement to acknowledge the skills, time and energy of those who are engaged.

Canada's variable and ad hoc approach to compensation creates complexity for organizations who must decide not only whether to compensate, but also how to compensate. This is contrasted by more standardized approaches to compensation in other jurisdictions, including guidelines released by the National Health Service in the United Kingdom and Australian consumer organizations.^19^, ^20^, ^21^ While standardized approaches to compensation can help streamline the process, they may forfeit the option for more individualized agreements between patient partners and partnering organizations. Flexibility in compensation may be important to meet patient partners' preferences for certain options like conference registration and to accommodate individual tax and income circumstances.

As Canadian organizations seeking to engage patients continue to evolve their practices around compensation, understanding the perspectives of patient partners seems important. However, limited research on this currently exists. Frameworks for ‘meaningful’ patient engagement and literature reporting on partnerships between research groups and patients suggest that appropriate compensation is a key facilitator of inclusive, equitable and desirable engagement.^22^, ^23^ A small group of patient partners published an instructive paper in 2018 on the ‘why’ and ‘how’ of compensation in research and health care.^10^ They suggest that compensation can help reduce power imbalances between patient partners and other team members by ensuring that everyone around the table is being paid. Compensation may also widen participation by enabling those who cannot afford to volunteer, thus promoting greater diversity within patient partnership.

Overall, the existing literature provides important but limited insight into patient partner views on compensation that may not reflect perspectives from the larger community of Canadian patient partners. As partnership and compensation practices continue to advance, it is important to consider what ‘appropriate compensation’ means to those individuals being engaged. Additionally, variability in rates and types of compensation make it difficult to know how patient partners are compensated in Canada, and whether current approaches meet the needs and preferences of patient partners. We sought to expand the scope of the existing literature to provide a more comprehensive view of Canadian patient partner perspectives on compensation by asking what types and frequency of compensation have patient partners been offered, and what are their attitudes towards compensation in general?

## 2 METHODS

### 2.1 Survey development and recruitment

This study uses data from the Canadian Patient Partner Study (CPPS) survey, an anonymous Internet‐based survey that ran from October to December 2020. Methodological details have been previously reported.^24^ The purpose of the survey was to understand the sociodemographic characteristics, activities, motivations, experiences, skills and challenges experienced by those who self‐identify as patient partners working across the health system in Canada. For the purposes of survey eligibility, we defined ‘patient partners’ as ‘people (patients, clients, family members and caregivers) who are drawing on their past or current experiences with the health system in some way, usually through their involvement in the activities of a particular health system group, organization, or government’. Given our interest in health system organizations and governments, patient partners working only in health research or in direct care were not eligible to participate. Eligibility for the survey was self‐determined and self‐reported.

The survey was developed in a multistep process beginning with a thorough literature review of existing surveys, followed by extensive consultations with the research team consisting of patient partners, an engagement practitioner and academic researchers.^25^ The survey was co‐designed with members of the Patient Advisors Network, with two co‐authors of the current paper serving as leads and full members of the research team. Additional feedback was sought from an external advisory committee consisting of patient partners and engagement practitioners working in Canadian and international contexts. The survey was pilot tested with 11 patient partners who worked across different Canadian health system settings in English and French. Pilot testing helped ensure the survey was accessible, comprehensive and captured the type of experiences we sought.

Formal recruitment for the finalized survey followed an online, chain referral approach, promoted by email and through social media.^26^ The first round of emails was sent to the project team and external advisory committee. These individuals were then requested to distribute the survey through their respective networks. Explicit steps were taken to identify and reach out to individuals or organizations with connections to historically underrepresented groups. The survey was also promoted on Twitter (now X), Facebook and LinkedIn multiple times over the recruitment period. The rationale behind the chain referral approach was to gather respondents with a diversity of perspectives, experiences and roles.

The survey was administered with LimeSurvey in both English and French.^24^ It was available online only with no paper‐based option. The survey consisted of 60 questions using Likert scales and drop‐down menus to capture discrete variables, plus open‐text comments. The study received ethics approval from the Hamilton Integrated Research Ethics Board (HIREB, #10705) and all respondents provided written informed consent before completing the survey.

### 2.2 Compensation‐relevant questions

**Figure 1:**
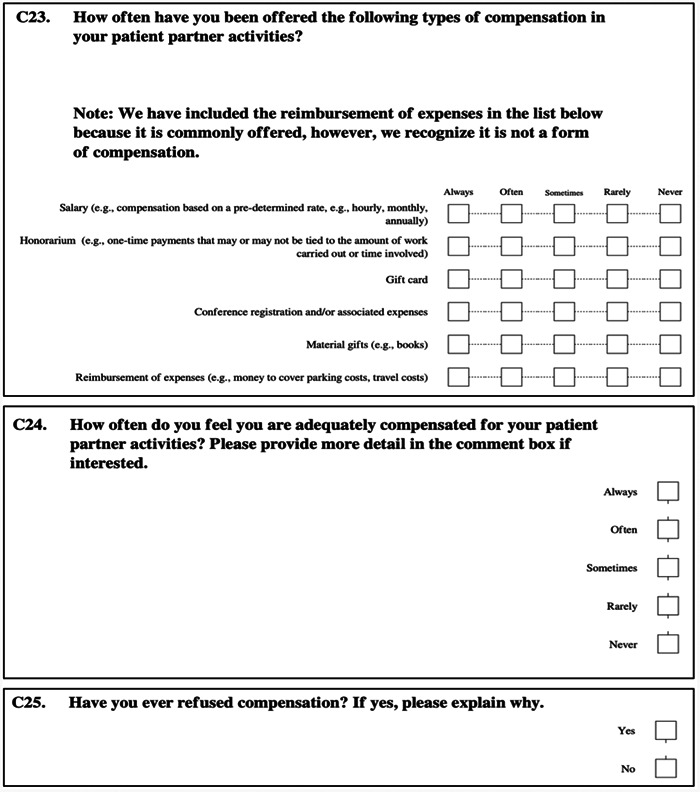
Compensation‐relevant questions from the Canadian Patient Partner Study survey.

Three questions in the survey focused specifically on compensation (Figure 1). The first sought to determine the various approaches used to compensate patient partners, asking ‘How often have you been offered the following types of compensation?’. Respondents could answer from ‘never’ to ‘always’ for each of the following: salary, honorarium, gift card, conference registration and associated expenses, material gifts and reimbursement of expenses. We included ‘reimbursement of expenses’ with a note that this isn't considered compensation but is commonly offered by organizations.

The next two questions sought to illuminate patient partner attitudes about compensation. Respondents could answer from ‘never’ to ‘always’ to the question: ‘How often do you feel you are adequately compensated for your patient partner activities?’. The final question asked, ‘Have you ever refused compensation?’ with a Y/N response. Both of these questions also allowed respondents to enter open‐text comments if they wished.

### 2.3 Data analysis

We analyzed the responses to all three survey questions to determine the prevalence of different approaches to compensation, adequacy of compensation and rates of refusal. Simple frequencies were calculated in Excel.

For analysis of open‐text comments, we used inductive (conventional) qualitative content analysis.^27^ Categories describing patient partners' rationales for supporting or opposing compensation were identified inductively from the open‐text comments in an initial round of coding of a subset of the sample by the lead author (R. D.). This initial schema was discussed and agreed upon by all team members before being applied to the entire data set. Codes were refined, modified, and consolidated as necessary, yielding the final schema outlined in Table 1. Once this schema was finalized, R. D. recoded the entire data set and another member of the research team (L. T.) verified all coding. Disagreements were resolved through discussion.

**Table 1:** Coding categories for qualitative analysis of open‐text comments.

Rationale	Description
Role‐based rationale	Patient partners attitudes towards compensation are tied to their role (e.g., volunteer, consultant/expert).
Volunteer/altruism‐based rationale	Patient partners attitudes towards compensation are tied to their identity as a volunteer and broader sentiments to improve or give back to the healthcare system.
Acknowledgement‐based rationale	Patient partners attitudes towards compensation are tied to how they feel valued and appreciated.
Instrumental rationale	Patient partners attitudes towards compensation are tied to the practical benefits or drawbacks of compensation (e.g., tax implications).
Fairness‐based rationale	Patient partners attitudes towards compensation are tied to principles of fairness and equity.
Accountability rationale	Patient partners attitudes towards compensation are tied to the accountability the patient partner has towards the organization, or the accountability the organization has towards the patient partner.

## 3 RESULTS

A total of 603 individuals participated in the CPPS survey. Eighty‐four percent of respondents were white, 77% were female and 83% were born in Canada. Almost 90% lived in urban areas and almost half reported having a chronic illness. Full demographic information about the sample is reported elsewhere.^24^

Over 80% of respondents answered the questions regarding type of compensation (Table 2). Overall, most respondents were never or rarely offered salary (81%), honorarium (64%), gift cards (80%) or material gifts (93%) in their role as a patient partner. Conversely, most individuals reported being offered reimbursement for expenses at least sometimes (80%). Being offered conference registration and associated expenses was more variable.

**Table 2:** How often respondents reported being offered each type of compensation.

Type of compensation	Total	Never/rarely (%)	Sometimes (%)	Often/always (%)
Salary	549	444 (81)	50 (9)	55 (10)
Honorarium	559	355 (64)	125 (22)	79 (14)
Gift card	531	425 (80)	82 (15)	24 (5)
Conference registration and/or associated expenses	526	263 (50)	148 (28)	115 (22)
Material gifts	511	475 (93)	28 (5)	8 (2)
Reimbursement of expenses	543	118 (22)	120 (22)	305 (56)

**Figure 2:**
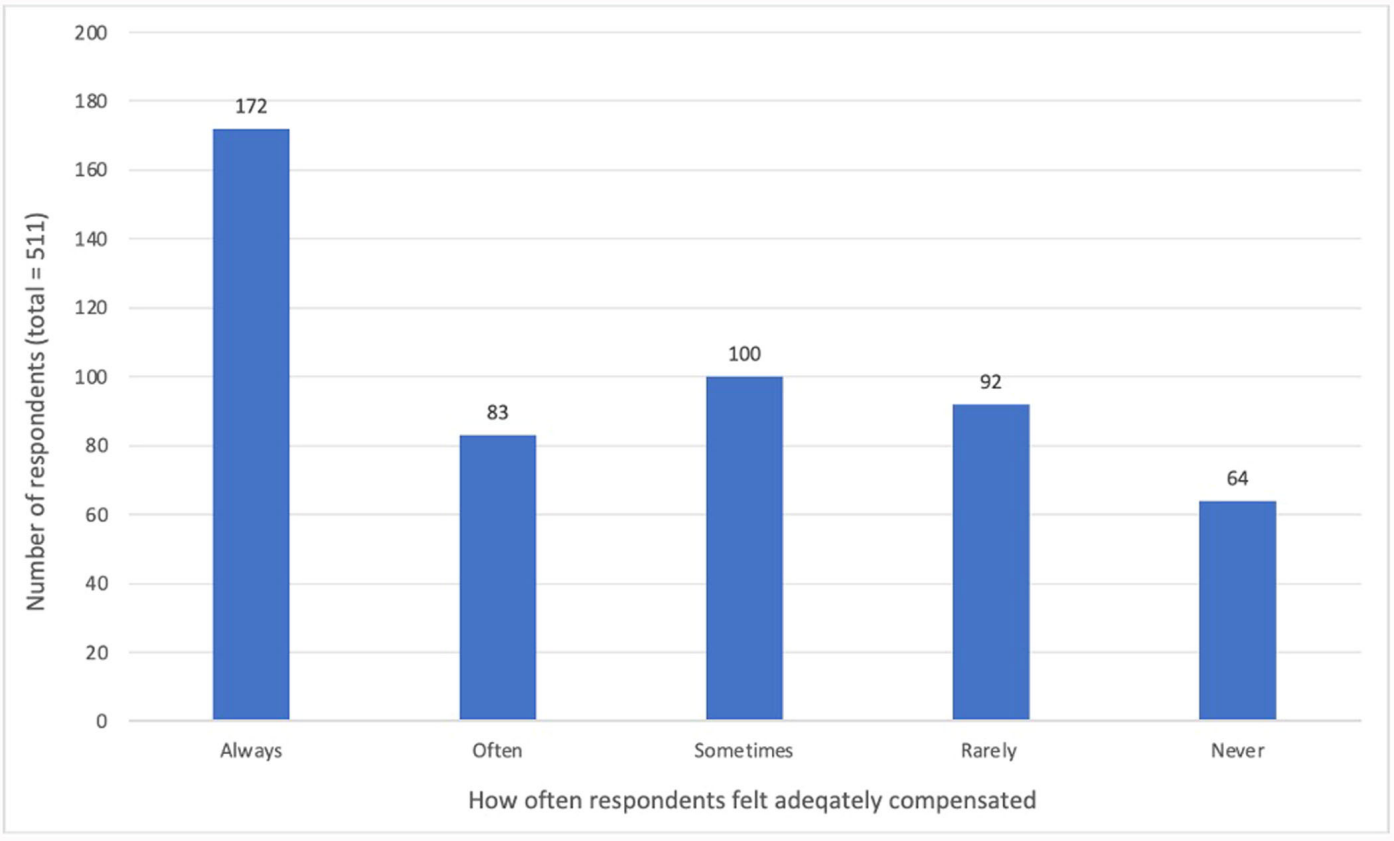
Number of respondents that felt adequately compensated always, often, sometimes, rarely or never.

A total of 511 individuals responded to the question asking if they felt adequately compensated (Figure 2). Of these, 34% reported always feeling adequately compensated and 12% reported never feeling adequately compensated. Between these poles, roughly equal proportions of respondents described feeling that they were adequately compensated often (16%), sometimes (20%) or rarely (18%). Of the 499 individuals who answered the question regarding refusal of compensation, 129 (26%) answered ‘yes’ to ever refusing compensation.

### 3.1 Attitudes towards compensation

A total of 364 individuals added an open‐text comment for the question regarding how often they felt adequately compensated and 216 individuals did so for the question asking if they had ever refused compensation. Individuals who left open‐text comments were more likely to indicate they never, rarely or only sometimes felt adequately compensated (55%) compared to those who did not leave open‐text comments (41%) (*p* = .002). The majority of respondents (69%) across both questions expressed attitudes towards compensation (support, ambivalence or opposition) which fit into one or more of the rationale‐based coding categories (Table 3). Within each rationale, attitudes towards compensation varied greatly. Some comments contained multiple rationales and were coded for each rationale separately.

**Table 3:** Summary of open‐text comments.

Rationale	Description	Number	Example quote
Role‐based rationale	Patient partners attitudes towards compensation are tied to their role (e.g., volunteer, consultant/expert).	140	‘I feel that patient advocates/advisors provide expert perspectives on par with medical professionals, yet are not compensated on the same level’.
Volunteer/altruism‐based rationale	Patient partners attitudes towards compensation are tied to their identity as a volunteer and broader sentiments to improve or give back to the healthcare system.	103	‘I am at a stage in my life where I am more interested in giving back to the community and sharing my knowledge. I do not need the money ‐ although I understand others have different positions and needs’.
Acknowledgement‐based Rationale	Patient partners attitudes towards compensation are tied to how they feel valued and appreciated.	100	‘The lack of remuneration reflects the lack of respect for this role’.
Instrumental rationale	Patient partners attitudes towards compensation are tied to the practical benefits or drawbacks of compensation (e.g., tax implications).	71	‘[Inadequate compensation] is a significant barrier to participation as I have to take time off my paid work to participate’.
Fairness rationale	Patient partners attitudes towards compensation are tied to principles of fairness and equity.	53	‘If [you] always leave it to volunteers, you will skew heavily to a certain demographic and miss essential voices around the table’.
Accountability rationale	Patient partners base their attitude towards compensation based on the accountability the patient partner has towards the organization, or the accountability the organization has towards the patient partner.	29	‘As an advisor, I am not interested in being paid for anything other than reimbursement of expenses. [I] want to be independent from the organizations I'm associated with’.

### 3.2 Role‐based rationale

The majority of respondents based their rationales about compensation on the nature of their perceived role within the organization. Respondents who perceived their role to be that of a volunteer were generally not looking for compensation outside of reimbursement of expenses: ‘I'm not seeking any compensation—I view myself as a volunteer’. However, many respondents who identified as volunteers felt an external pressure to contribute without compensation even though they may have desired it: ‘usually the expectation is that it is volunteer work; even when working in my professional field giving training as a patient partner, I am expected to do it for free’.

Respondents who desired compensation generally saw their role as going beyond casual volunteerism, invoking self‐assessments of expertise in relation to lived experience. ‘There is a disconnect between the concept of patients as full partners who are experts with lived experience and the way they are generally compensated as volunteers’. Offering compensation that was deemed inadequate, especially when compared to other professionals, led to dissatisfaction: ‘Many organizations offer such a token amount that it really is demeaning. In my professional life my “brain” is worth much more, but when I come to the table as an expert patient advocate with years of experience, I am offered an amount so low that it is actually costing me money to participate!’

Further comparisons between patient partners and other professionals evoked feelings of unfairness and inequity in some respondents: ‘I understand that my position is a volunteer position. However, it sometimes irks me to know that of those sitting around the table at a meeting (or during a virtual meeting) only the patient partners participating are not being paid. Everyone else around the table is paid to be there’.

### 3.3 Altruism‐based rationale

A significant number of respondents, especially those who noted a preference for volunteer roles, tied their views on compensation to a desire to give back to the health system: ‘My “payment” is the betterment of the patient experience, for me and others’. ‘I do not expect compensation. I do it because there is a need for change and improved care’. Respondents who accepted or desired volunteering generally opposed or were ambivalent towards compensation: ‘I do not want money spent to compensate me for my volunteer work since I am trying to improve a health system that is always in need of funds’.

Further, respondents who saw themselves as volunteers saw important distinctions between themselves and paid professionals: ‘As a volunteer I don't expect money. I realize professionals are paid for the same time we are together. But I can choose not to be there they can't’. Such distinctions may explain the lack of comments in this section alluding to fairness issues between patient partners and other paid members.

However, some comments in this section expressed tensions and potential tradeoffs within the volunteer role: ‘I want to help make a difference sharing our experiences and I don't do it to get paid. However, I cannot do as much as a family advisor as I would like because I need to focus on my paying job’. Thus, while volunteering may be desired, it may be impractical for some patient partners: ‘We as patient partners would do this for free to share our lived experiences to situations so they will improve and change for the better, but most of us [are] on a limited budget and extras would be very appreciated’.

The following comment captures the tensions inherent in patient partner attitudes towards compensation and the interplay of individual‐level factors that influence the desire to be compensated or not: ‘I do it because I feel it is the right thing do it, because I enjoy it, my circumstances can afford it and many other reasons, but they [the organization] are getting access to an expert and using my contributions for no pay’.

### 3.4 Acknowledgement‐based rationale

Within this category, respondents either saw compensation as an important way to appreciate, value and respect patient partners or not—we have termed this appreciation as ‘acknowledgement’. Respondents using the acknowledgement‐based rationale viewed financial compensation as either essential, supplementary or unnecessary to feeling acknowledged.

For quite a few respondents, compensation was essential to feeling valued in their role. They viewed their time, skills and expertise as deserving formal compensation. Others saw it as an important sign of respect from the organization without which patient partnership is devalued: ‘Our time was initially compensated on an hourly rate that was respectable and allowed for preparation/pre reading and transportation. It's since been reduced to honorarium and is significantly lower, so that our time and contribution are devalued and disrespected even as the language and platitudes have become more lofty’.

For others, being compensated was not necessary to feel appreciated and valued: ‘I assume you mean financially compensated. That is never a factor in making me feel my contributions were appreciated. That being said, I realize those are my personal feelings and appreciate that others feel very differently and with good reason’. Exactly what ‘acknowledgement’ looks like outside of compensation is unclear but individuals who felt properly acknowledged said they were ‘treated with respect and regard’ and ‘made to feel [their] opinions matter’.

Finally, for some, compensation was not required but did foster a feeling of acknowledgement: ‘I do this work because I see value in it, not for the compensation. I appreciate that my time and input is worth something and appreciate being compensated when possible. I would often complete the activities on a voluntary basis even without compensation’. Interestingly, some individuals thought that the opportunity to partner and advance within the role was adequate reward for participation and formal compensation was only supplementary: ‘Reward has mostly been in the area of building capacity and growth in level of engagement opportunities’. Others felt that the value of the work was in being able to contribute positively to the healthcare system, a sentiment also echoed by those who wanted to volunteer: ‘The reward comes in doing the work, learning the issues and the occasional good outcome. The price of admission is eternal optimism that we can collectively improve the system’.

### 3.5 Instrumental rationale

Respondents who discussed compensation using an instrumental rationale appealed to the more practical aspects of compensation, like requiring compensation to feasibly engage in patient partnership or refusing compensation to avoid tax liabilities. Respondents who highlighted the importance of compensation often suggested that ‘without compensation I would not be able to do the work that I do’ and that inadequate compensation ‘is a significant barrier to participation as I have to take time off my paid work to participate’. Many respondents discussed the costs associated with having the tools to participate: ‘having a computer and printer (with ink) are so great, I have to defray these costs in some way’. Despite reimbursement of expenses not being considered a form of compensation, respondents identified costs of participation (e.g., travel) as a significant barrier and frustration.

Often, recipients of government income supports, such as disability support payments, were required to refuse compensation as it would have a negative impact on their ability to retain disability support, with one respondent writing that, ‘[I] can't accept any monetary compensation as I am on disability and would have to have this income deducted on a dollar for dollar basis’. However, they go on to say that ‘It would [be] great however to receive gift cards or assistance with technology required to do this work’, suggesting that other forms of compensation may be desirable. Overall, while many in this category reported refusing compensation, this was more so to comply with tax policies, rather than a desire to volunteer or forego compensation entirely.

Some respondents were ambivalent about receiving compensation. Many in this group specifically noted that they were retired and compensation wasn't something they were looking for. However, several in this category still noted relying on volunteers is often unsustainable, inequitable and exclusionary: ‘I think there should be more compensation. I am older and am lucky to be financially stable, but there are many not at the table because they have full time work’. These comments also combined the instrumental rationale with the fairness rationale to advocate for compensation: ‘Patient partners are never compensated adequately for the work they do—this limits patient advisors to a very small group of people and are not representative of all parts of our community, especially the most vulnerable’.

### 3.6 Fairness rationale

Respondents who invoked principles of fairness and equity in their comments expressed mostly supportive attitudes towards compensation. A number of respondents acknowledged significant inequity in groups where patient partners and other professionals worked together: ‘I attended 60+ meetings of the working groups for free. I found out afterwards all the doctors who participated were given generous stipends for attending’. Individuals who saw themselves as contributing expert knowledge in a comparable way to professionals found such inequities in compensation especially problematic, ‘Patients are expected to provide their specialized knowledge for free to the health care system. Altruistic while all others at the table are paid. This is incredibly destructive to the power dynamic, and promotes inequality in the discussion’.

The type of compensation received also shaped respondents' perceived inequities between patient partners and other professionals. As one patient partner notes, ‘It does feel weird to receive a gift card when you know that other speakers at the same conference were highly paid’.

Other comments discussed compensation as a way to promote equity within patient partnership: ‘I am financially well off and do not seek compensation. I am retired and have the time and passion to be involved. However, there are other people who are not as fortunate as I am and financial consideration to include their participation is necessary. Otherwise, the perspective of people of lower socio‐economic status is not reflected in the data collected in my opinion’.

One respondent noticed a difference in their experience with partnership when they have been compensated: ‘I don't expect compensation but in cases where it has been provided I have been treated as more of an equal and the role is taken seriously rather than a token member’.

### 3.7 Accountability‐based rationale

A small number of respondents expressed their views about compensation through an accountability lens either referring to the responsibility of patient partners to the organization or vice versa. Those who opposed compensation were wary about its influence on self‐censorship: ‘I worry that with compensation… I would be forced to focus on their priorities rather than being the true, unfiltered voice of the community lived experience and I have seen this happen far too often’.

Conversely, others supported compensation as an important way for organizations to be accountable to patient partners: ‘Compensation would demonstrate a complete/genuine commitment by [organization] to the enhancement of healthcare and inclusion within our system. It would put their money where their mouth is, so to speak’. One respondent noted that monetary expense may lead to greater commitment to impactful engagement: ‘When orgs/govs/researchers have a line item for the progress and results of a group of people, they are more keen to produce and demonstrate results’.

### 3.8 Other experiences with compensation

While this analysis focuses on patient partners rationales for supporting or opposing compensation, several comments in our survey described issues in the process of requesting and receiving compensation that was promised by organizations. Respondents often felt that the onus of initiating the conversation about compensation was solely on them, and many mentioned feeling awkward, ashamed and humiliated by these conversations: ‘[Organization] makes a big deal about being compensated. You almost feel embarrassed if you take compensation—even for mileage!’

Some also pointed to the burdensome process of having ‘to fight [for] even small amounts of compensation’: ‘I have worked on an engagement where they were to reimburse my travel and there was such low engagement with the team that they didn't even fulfil the promise. I felt petty going after bus fare but it really says something to me when they can't even do that’. Finally, even when receiving compensation, one respondent reported being afraid of billing accurately and how it might be perceived by the organization: ‘I sometimes get paid, but when I do it is an hourly rate. I'm concerned my bill will be too much and so I don't count all my hours. I don't want them to stop involving me’.

## 4 DISCUSSION

The results of this study provide important insights into Canadian patient partners' experiences with and perspectives on compensation. While previous papers and resources have identified the importance of compensation and addressed the ‘how to’,^10^, ^11^, ^12^, ^13^, ^14^, ^15^, ^16^ to our knowledge, this is the first analysis encompassing the views of a wide range of patient partners across Canada. Our survey confirms that compensation of patient partners is not a standardized or consistent practice in Canada, with patient partners reporting being offered compensation of varying types with varying regularity. Interestingly, perceptions about adequacy of compensation were split amongst our respondents, with half of patient partners routinely feeling adequately compensated often or always and half feeling that compensation is only sometimes, rarely or never adequate. This dichotomy reverberates in the tensions respondents expressed between unpaid and paid roles and attitudes towards compensation that were shaped by their personal circumstances (e.g., retirement status, disability status, desire to volunteer) and broader issues in patient partnership (e.g., power dynamics, equity, fairness and accountability).

While we explicitly stated in our survey that reimbursement of expenses is not a true form of compensation, we included reimbursement as a response option at the advice of patient partners who noted that it is the most common form of ‘compensation’ offered; this advice held true in the data. However, consistency in reimbursement and challenges in the reimbursement process persist. While some expenses may be covered on a regular basis (e.g., travel, parking), many respondents noted that they still incur costs for other, less commonly considered, expenses such as home printing, technology or internet access. These challenges were likely exacerbated during the time of the survey, as many engagements had moved online due to the COVID‐19 pandemic.^28^ Additionally, many respondents highlighted issues in asking for reimbursement, as organizations often put the onus of requesting reimbursement, as well as navigating complex billing systems, on the patient partner.

The type of compensation offered has important implications for patient partners. Our survey suggests that some patient partners may only be able to accept certain forms of compensation. Many report having to refuse compensation due to tax implications or fear of losing their disability status. However, some respondents also mentioned that they would have accepted an alternative, suggesting that refusal of compensation does not necessarily reflect a lack of need or desire to be compensated. Organizations should consider how patient partners' unique contexts may make receiving certain forms of compensation undesirable or impossible. While this perspective supports more flexible compensation packages as seen in existing Canadian organizational guides,^16^, ^17^, ^18^ organizations should also be mindful that self‐advocacy for appropriate compensation requires self‐efficacy and the ability to navigate often intimidating organizational bureaucracy. Organizations may attempt to ameliorate this burden by initiating conversations with patient partners, clearly describing the available compensation options, and making processes to apply for and receive compensation as straightforward as possible.

We find an important link between compensation and equity in our survey. Equity amongst patient partners and between patient partners and other professionals was of significant concern to our respondents regardless of personal preference for volunteering. Those who did not need or want compensation still recognized the importance of promoting a culture of fair compensation for patient partners. This perspective is consistent with the broader discourse of equity, diversity and inclusion (EDI) in patient partnership where compensation is often mentioned as an important (but insufficient) element to advancing EDI.^29^, ^30^, ^31^, ^32^ Compensation contributes to ensuring that patient partnership is not limited to those who can afford to volunteer their time and can offset at least some of the socioeconomic barriers to getting involved as a patient partner.^23^, ^33^, ^34^ Partnership initiatives which fail to consider how social location (e.g., income, gender, race) impact one's ability to expend resources to participate in patient partnership often end up engaging the most ‘easy to reach’ individuals—those who are ‘white, well‐educated, and well‐resourced’.^32^ This may be reflected in our relatively homogeneous survey sample of mainly white cisgender women over age 50 of high socioeconomic status.^24^

Overall, our survey offers some important insights for organizations seeking to ensure that all patient partners can receive adequate compensation while still accounting for individual needs and preferences. First, organizations must attempt to provide fulsome reimbursement of expenses to ensure that patient partners do not incur any personal costs for engagement. The process should be as streamlined as possible to reduce burdens, both time and effort and emotional, on patient partners. As recommended by others in the field, our survey suggests that organizations should initiate clear and transparent conversations about compensation at the outset of partnerships.^10^, ^11^ This conversation should discuss in advance both a partner's desire and ability to accept different types of compensation and the organization's budget. While an individual and institutional fit may not always be possible, initiating the conversation can ensure that patient partners not only feel heard and respected in the process, but can also reduce the anxiety felt by patient partners in the absence of overarching standards for compensation that could frame expectations. Finally, organizations should bear in mind the equity implications of compensation. This includes considering how compensation for patient partners may fare against other professionals who are part of the engagement process, and which voices will be systematically excluded due to a lack of adequate compensation.

## 5 LIMITATIONS

This study is limited to the views of patient partners who participated in the CPPS survey, a demographically homogeneous group. In particular, our respondents were overwhelmingly white, female, cisgender and born in Canada. This response pattern may indeed reflect the broader population of patient partners but could also be a consequence of our online recruitment method, which may have favoured more technologically adept respondents who are well‐connected within patient engagement networks across Canada. Notably, our recruitment method may not have reached individuals who self‐identify as patient partners but do not belong to formal and informal patient partner networks. We may have also missed individuals lacking the technological knowledge or resources to complete the survey online.

Since there is no existing database of patient partners, we cannot determine how well our survey reflects all individuals who may self‐identify as patient partners in Canada. Additionally, eligibility was self‐reported, so it is possible that some people who were not eligible still completed the survey. Eligibility was determined on the basis of responses, corresponding with a definition of ‘patient partner’ and exclusion criteria that were clearly stated at the beginning of the survey. Responses indicating that a participant was not eligible were not able to continue to respond to further questions in the survey. Since our survey is limited to the views of patient partners and compensation is one of many equity barriers to entry for would‐be patient partners, our survey cannot represent the views these individuals may have towards compensation. Finally, as this study is focused on the perspectives and experiences of patient partners, we cannot address institutional and administrative barriers that organizations face when compensating patient partners. This should be the focus of future work.

## 6 CONCLUSION

This research provides the first wide‐ranging analysis of patient partners' experiences of and perspectives on compensation from a large pan‐Canadian survey. Our findings suggest that patient partners base their attitudes on how compensation intersects with (1) their perceived role, (2) a desire to give back to the health system, (3) feelings of acknowledgement and appreciation, (4) barriers and facilitators of engagement, (5) equity and fairness and (6) accountability of patient partners and partnering organizations. While some patient partners may not want or be able to receive compensation, often due to personal circumstances, promoting a culture of compensation is seen as an important facilitator of equity and inclusion in patient partnership. These perspectives shed light on how patient partners would like organizations to approach compensation: as an adaptable practice that is sensitive to patient partners' individual contexts, informed about the demands of patient partnership and attentive to the broader movement towards greater equity and accessibility of patient partnership.

## AUTHOR CONTRIBUTIONS

**Roma Dhamanaskar**: Writing—original draft; methodology; formal analysis; writing—review and editing; validation; visualization; data curation. **Laura Tripp**: Writing—original draft; methodology; validation; formal analysis; project administration; data curation; investigation. **Meredith Vanstone**: Supervision; conceptualization; investigation; funding acquisition; writing—review and editing; methodology; validation; formal analysis. **Carolyn Canfield**: Writing— review and editing; investigation. **Mary Anne Levasseur**: Writing—review and editing; investigation. **Julia Abelson**: Investigation; conceptualization; supervision; writing—review and editing; methodology; validation; funding acquisition; formal analysis.

## CONFLICT OF INTEREST STATEMENT

The authors declare no conflict of interest.

## ETHICS STATEMENT

This research received ethics approval from the Hamilton Integrated Research Ethics Board (#10705). All participants provided informed consent.

## Data Availability

The data that support the findings of this study may be available from the corresponding author upon reasonable request.
